# Mental disorders in foster children: a study of prevalence, comorbidity and risk factors

**DOI:** 10.1186/1753-2000-7-39

**Published:** 2013-11-21

**Authors:** Stine Lehmann, Odd E Havik, Toril Havik, Einar R Heiervang

**Affiliations:** 1 Department of Clinical Psychology, Faculty of Psychology, University of Bergen, Cristiesgate 13, Bergen, 5015, Norway; 2 Uni Research, Uni Health, Regional Centre for Child and Youth Mental Health and Child Welfare, Bergen, Norway; 3 Institute of Clinical Medicine, University of Oslo, Oslo, Norway; 4 Regional Office for Children and Family Affairs, Region South, Norway

## Abstract

**Background:**

The aim of this study is to examine the prevalence of mental disorders in 6- to 12-year-old foster children and assess comorbidity and risk factors.

**Methods:**

Information on mental health was collected from foster parents and from teachers using Developmental and Well-Being Assessment (DAWBA) Web-based diagnostic interview. Child welfare services provided information about care conditions prior to placement and about the child’s placement history.

**Results:**

Diagnostic information was obtained about 279 (70.5%) of 396 eligible foster children. In total, 50.9% of the children met the criteria for one or more DSM-IV disorders. The most common disorders were grouped into 3 main diagnostic groups: Emotional disorders (24.0%), ADHD (19.0%), and Behavioural disorders (21.5%). The comorbidity rates among these 3 main groups were high: 30.4% had disorders in 2 of these 3 diagnostic groups, and 13.0% had disorders in all 3 groups. In addition, Reactive attachment disorder (RAD) was diagnosed in 19.4% of the children, of whom 58.5% had comorbid disorders in the main diagnostic groups. Exposure to violence, serious neglect, and the number of prior placements increased the risk for mental disorders.

**Conclusions:**

Foster children in Norway have a high prevalence of mental disorders, compared to the general child population in Norway and to other societies. The finding that 1 in 2 foster children presented with a mental disorder with high rates of comorbidity highlight the need for skilled assessment and qualified service provision for foster children and families.

## Background

In Western societies, the number of children placed out of home converged at approximately 5 per 1000 in 2006-2007 [1]. In Norway [2], as in most western societies [3], parental neglect endangering a child’s development and health is the primary reason for out-of-home placement, and families receiving services from the child welfare system are often characterised by low socioeconomic status [4]. Child welfare services in Norway are typically family-oriented, emphasising voluntary and preventive home-based interventions. After a family’s first contact with child welfare services, children continue to stay, on average, 3 years with their biological families receiving home-based services, before they are placed out of home [5]. However, once the child has been placed in a foster family, the placements tend to last longer than in Anglo-American countries [3].

The prevalence of mental health problems in foster children has primarily been investigated using symptom checklists, providing an overall estimated prevalence of mental health problems in the range of 42.7% to 61.0% [6-11]. Because questionnaires do not allow for detailed enquiry into symptom patterns, duration, or functional impact, these estimates may not be equated with estimates based on diagnostic assessments. Furthermore, symptom checklists do not take into account comorbidity rates.

Standardised diagnostic interviews are seen as the best way to achieve reliable prevalence estimates for mental disorders in different populations. However, only a few studies so far have used such diagnostic interviews to estimate the prevalence of mental disorders among foster children. One early study reported a point-prevalence of DSM-III-R disorders of 57.0% in foster youth [12]. A rather similar overall prevalence rate of 50,0% has been found in a more recent study of foster youth aged 11-17 years. [13] McMillen et al. [14] reported a somewhat lower past-year prevalence of 33.0% in a comparable sample, with 17.0% having Conduct Disorders (CD) or Oppositional Defiant Disorder (ODD), 15.0% Major depression, and 10.0% Attention Deficit Hyperactive Disorder (ADHD). Consistent with other studies, [12,15,16] the prevalence was higher for youths placed in congregate care [14]. In a study of foster youths aged 17 years and older, Keller, Salazar and Courtney [15] reported a lifetime prevalence of DSM-IV disorders of 10.5% for Major Depression and 16.1% post-traumatic stress disorder (PTSD).

These interview-based diagnostic studies all assessed older foster youths, using self-report only. The only sample that included younger foster children was the study by Ford, Vosansis, Meltzer and Goodman [16]. They reported a point-prevalence of 38.6%, where 9.7% suffered from Emotional disorders, 32.3% had CD/ODD and 8.5% had Hyperactivity. In this study, the diagnostic information was obtained from teachers, caregivers, and youths from 11 years of age. A higher prevalence rate was found in boys than in girls, and the rates increased with age. Whether this age-related increase could be attributed to later placement and longer exposure to neglect and abuse was not explored. Furthermore, the prevalence was only reported for broader diagnostic groups and not for single disorders among children living in foster families.

In contrast to the general agreement regarding the diagnostic criteria and methods of assessment for most mental disorders in children, the validity and relevance of the criteria for the diagnosis of Reactive Attachment Disorder (RAD) have been more controversial, especially regarding how these features should be characterised and assessed after the age of 5 years old [17,18]. Some longitudinal studies have continued to use the Strange Situation Procedure up until school age, in combination with parental reports and standardised investigator ratings of child behaviour [19,20], while others have developed their own semi-structured interviews and rating scales [21].

Findings indicate that children exposed to early adverse childhood experiences in general [22] and more specifically children placed in foster care have a heightened risk of attachment difficulties [23,24]. Further, attachment difficulties have been related to other mental health problems both among foster and adopted children [25,26]. It is therefore important to include measures of attachment disorders when assessing mental disorders of foster children.

Recently, a RAD section was added to the Developmental and Well-Being Assessment (DAWBA) structured diagnostic interview manual [27], developed from the corresponding section of the Child and Adolescent Psychiatric Assessment interview [28]. The first study using the DAWBA-RAD section reported a very high RAD point-prevalence of 63.0% (96/153) in a sample of looked after youth in a variety of placement forms [29]. In this study however, RAD was not defined according to the Diagnostic and Statistical Manual of Mental Disorders (DSM-IV) [30] criteria, but as a symptom score 2 standard deviations greater than the mean. There is therefore a need for further studies of the prevalence of RAD among school-aged children living in foster families, as this age range and placement form are the most common in child protection services.

Age, sex, and learning difficulties [31,32], as well as low socioeconomic status [33,34], are well-established predictors of mental health problems in children in general. Foster children are exposed to a range of other risk factors as well [35]. Adverse childhood experiences, such as psychological and physical abuse and neglect, parental substance abuse and mental illness, all increase the risk of both physical and mental health problems, as well as health risk behaviours [36-41]. In addition, older age at placement, frequent placement changes, the number of placements and persistent adverse events after placement pose additional risks for these children [42,43]. However, few studies so far have examined whether such risk factors show specific associations with certain types of mental disorders [44].

In summary, previous studies have converged on the finding that foster children represent a high-risk group for mental health problems and that these problems might be associated with experiences of early neglect, abuse, and other adverse childhood experiences. However, only a few studies have used diagnostic interviews, covering the full range of mental disorders, and only one of these studies included school-aged foster children who were still living in foster families.

The purpose of this study was to estimate the point-prevalence and comorbidity of DSM-IV disorders in school-aged foster children. Further, we aimed to investigate the predictive value and specificity of risk factors related to adverse childhood experiences prior to placement, and placement history with regard to mental disorders in these children.

Because most foster children have been exposed to neglect and abuse before placement, we expected them to show increased rates of mental disorders compared to the general population [32]. We expected greater exposure to risk factors to be related to a higher prevalence of mental disorders, and in line with existing research findings, we expected that psychological and physical abuse, parental substance abuse and mental illness in the family of origin would be positively associated with mental disorders. Further, we expected to find associations between the prevalence of mental disorders and an unstable placement history.

## Methods

### Sample: eligibility and recruitment

The inclusion criteria were children aged 6 to 12 years old, living in foster families encompassed by the Southern Regional Office for Children, Youth and Family Affairs for at least 5 months following legally mandated placement. According to records from the Regional Office for Children, Youth and Family Affairs, there were 391 eligible children living in the 63 municipalities of the region.

Informational letters were sent to the head of each municipal child welfare office. The office heads were asked to review the list from the regional register of foster children and to complete the list by adding eligible children who were not registered. This process led to the identification of 28 additional eligible children. Of the registered children, the municipalities reported that 20 had either returned to their biological families or had been adopted. Three additional children were deemed ineligible because of serious neurological disabilities. Thus, the final number of eligible children was 396. The child welfare offices in the municipalities were asked to provide contact information for these children’s schools and teachers. They were also asked to answer a short purpose-made questionnaire about the children’s care conditions prior to placement and their placement histories. The caseworkers did not provide any diagnostic information, so the diagnoses are based on the DAWBA from the foster parents and the child`s teacher.

**Figure 1:**
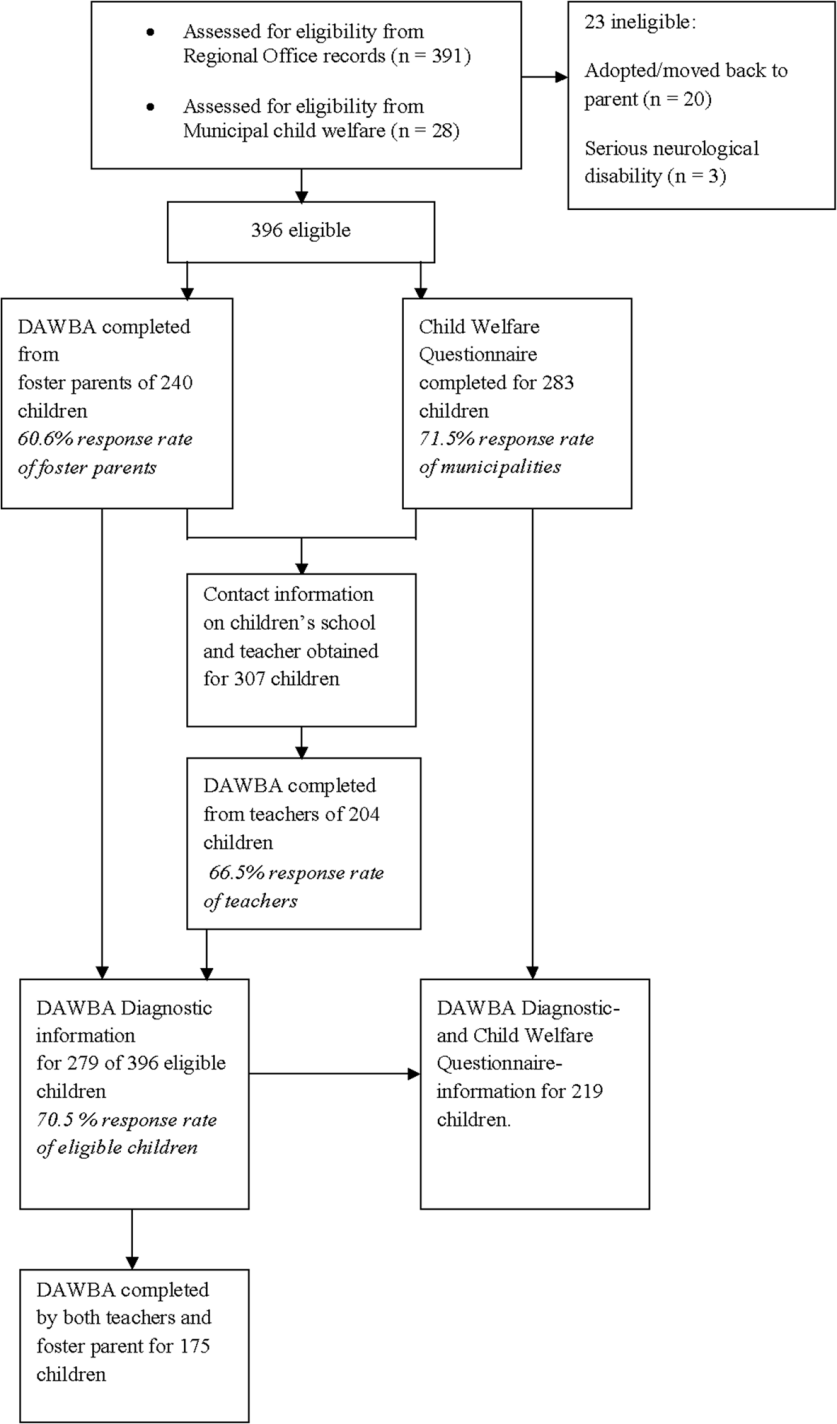
Flow-chart of data collection.

Foster parents of the 396 eligible children received postal letters with detailed information about the study, as well as instructions on how to complete the DAWBA interview online. They were also asked to return contact information for the children’s schools and teachers. In total, contact information was obtained for 307 teachers, who were then contacted by postal mail and asked to complete a version of the DAWBA diagnostic interview online. Figure 1 provides a flowchart of the entire data collection.

### Ethics

The study was approved by the Regional Committee for Medical and Health Research Ethics, Western Norway. The Ministry of Children, Equality and Integration provided exemptions from confidentiality for caseworkers, foster parents, and teachers participating in this study. In accordance with Norwegian ethics requirement, oral assent is required from children aged 12 years old. The children and their foster parents were instructed about this in the information letters that included a version especially adapted for children. If the child did not assent, the foster parents were instructed not to participate in the survey.

### Measurements and diagnostic rating procedures

We used the Developmental and Well-Being Assessment (DAWBA) [27] interview to assess DSM-IV mental disorders. The DAWBA is a Web-based diagnostic interview that combines structured questions on symptoms and impairment with open-ended questions in which the respondents describe the child’s problems in their own words. The DAWBA administered to parents or caregivers has a total of 17 sections, covering diagnostic areas, child and family background, and child strengths. The time needed to complete the interview by carers vary from 30 minutes to several hours, depending on the amount of problems reported. Due to skip-rules included in the web-based interview, the interview becomes shorter if no problems are reported in the initial questions of a section. Teachers respond to a shorter version of the interview, which typically can be completed in 15-30 minutes.

The task of the clinical rater is to judge the answers from the different informants. For most disorders, the diagnostic criteria only require that problems are evident in one setting (e.g. at home or at school). The different informants are usually treated as complementary adding to the understanding of the child. Where informants give contradictory information, the rater has to use her judgment as to witch informant is the most reliable. The DAWBA interview has shown good ability to discriminate between children from community and clinical settings [27], and it has generated realistic prevalence estimates of mental disorders when used in public health services [32,45].

In this study, all of the available DAWBA information from both foster parents and teachers were reviewed by first and last author, who separately assigned diagnoses according to the DSM-IV criteria. Both raters are clinical specialists in child and youth mental health. Last author has documented high inter-rater agreement with Robert Goodman, who developed the DAWBA [32]. The agreement between the 2 raters regarding the presence/absence of a disorder was excellent (Kappa = 0.95, 95% CI: 0.88-1.00).

If informants reported a definitive impairment in function but not sufficient symptoms to fulfill a specific diagnosis, an “other” or NOS diagnosis was given. A previously given ADHD diagnosis by a specialist in child mental health services was accepted, even if the ADHD interview section reported sub-threshold symptoms and impairment, because the symptoms might have been suppressed by medication. For children from the age of 11 years, the RAD section is not a part of the DAWBA interview. For the children aged 11-12 years old, we therefore used free-text description of symptoms and impairments meeting the DSM-IV criteria to assess RAD. A previously given RAD diagnosis by a specialist in child mental health services was also accepted for this age group.

A short child welfare questionnaire was developed for the study to obtain information from caseworkers in the child welfare services, about 12 possible care conditions in the biological family; the caseworker could mark any number of these conditions, corresponding to their records of characteristics of the child’s care experiences. The questionnaire also asked about placement history and the country of birth of both the child and biological parents.

### Procedures

The data collection started in September 2011 and lasted for 6 months. If foster parents or teachers had not responded within 2 weeks after the first information letter, a reminder was sent. Consenting foster parents who still had not completed the DAWBA within 2 months were offered a telephone interview. Thirty-one DAWBA interviews were completed over the phone. Teachers were compensated with NOK 250 (31 Euro) for their participation, while foster parents were not offered compensation for participating.

### Analysis

Statistical analyses were performed with the Statistical Package for the Social Sciences (SPSS), version 19 for Windows. Comparison between subsamples was performed with *t*-tests and Chi-square tests. The prevalence of disorders was calculated by frequency analyses with 95% confidence intervals (CIs). In subsequent analysis, single disorders were clustered into 3 main diagnostic groups. Due to the relatively low prevalence of depression, this disorder was grouped together with all of the anxiety disorders and with undifferentiated anxiety/depression in the main diagnostic group of Emotional disorders (see Table 1). Diagnoses related to ADHD were grouped into ADHD disorders. Similarly, CD, ODD, and other disruptive disorders were grouped into the diagnostic group of Behavioural disorders. This grouping of disorders corresponded to that used in Ford et al.’s study of looked-after children [16]. Further, the RAD group comprised only that diagnosis. The group labelled “Any disorders” comprised all single disorders referred to in Table 1, except for the NOS diagnosis.

**Table 1:** Characteristics of foster children with both DAWBA and municipal care history information (n = 219)

	**%**	**Mean**	**SD**
Age (years)		8.97	2.04
Female gender	47.0		
Former placements		0.90	0.85
0	32.0		
1	52.2		
2	12.5		
3-5	3.1		
Age at first placement		3.74	2.98
0–6 months	16.0		
7 months–2 years	26.0		
3–5 years	28.8		
6–12 years	29.2		
Years in current foster home		5.08	3.06
0-2	23.5		
3-5	25.3		
6-7	25.3		
8-12	25.8		
Number of adverse childhood experiences^1^		3.00	1.60
Violence exposure (range 0–4)^2^		0.71	1.14
0	64.1		
1-2	26.2		
3-4	9.7		
Serious neglect	86.3		
Parent`s drug/alcohol abuse	55.3		
Parent`s mental disorder	52.3		
Parent`s mental disability	9.6		

^1^Experiences in family of origin; ^2^Violence exposure = the sum of witnessing domestic violence; exposure to physical violence; exposure to emotional abuse; witnessing emotional abuse.

Cross-tabulations were used to examine patterns of comorbidity, first between each of the 3 main diagnostic groups — Emotional disorders, ADHD and Behavioural disorders — and all other disorders, and then among these 3 main diagnostic groups only. These 3 groups were further recoded into 1 variable to examine the overlap between RAD and any of these 3 main diagnostic groups. Estimates of the odds of comorbidity between any 2 of 4 diagnostic groups (Emotional disorders, ADHD disorders, Behavioural disorders, and RAD) were calculated with logistic regression analyses.

In the analyses of associations between possible risk factors and mental disorder, the 5 diagnostic groups (Emotional disorders, ADHD disorders, Behavioural disorders, RAD and Any disorders) were included as the dependent variables in separate binary logistic regression analyses. To reduce the number of predictors, the associations between single risk factors and diagnostic groups were examined in preliminary analyses (see Table 1 for information about the included predictors). Among the demographic variables, Age, but not Gender or Parents ethnicity, was related to at least 1 of the 5 diagnostic groups. Variables related to the child’s placement history (Age at first placement, Number of placements, and Time in current foster home) were all related to at least 1 diagnostic group. Time in current foster home and Age at first placement were highly inter-correlated (r = -0.69). To avoid collinearity, only Age at first placement was included in the subsequent analyses.

Among the possible risk factors reflecting care experiences in the family of origin, as reported by the child welfare services, Parental substance abuse, Mental illness and Mental disability were unrelated to any of diagnostic groups. Five variables — Serious neglect, Exposure to physical violence, Witnessing domestic violence, Exposure to emotional abuse (threats, hostility, rejection, harsh verbal punishment), and Witnessing emotional abuse towards other family members — all had a significant associations with at least one diagnostic group. These 5 variables were then included in an exploratory principal component analysis with oblimin rotation. The latter 4 of the 5 variables were loaded on one factor with an eigenvalue of 2.18, explaining 43.7% of the total variance, whereas Serious neglect was loaded as a separate factor, with an eigenvalue of 1.08, explaining 21.1% of the total variance. Based on these findings, the 4 variables loading on Factor 1 were added into a continuous variable termed Violence exposure, with a range of 0–4 (M = 0.89, SD 1.22) and Cronbach’s alpha = 0.72. Thus, in the final logistic regression analyses, the following predictors were included: Age; Age at first placement; Number of placements; Violence exposure; and Serious neglect. All of the predictors were used as continuous variables, except for Serious neglect, which was defined as a dichotomous variable (no = 0, yes = 1), using a simple contrast with no serious neglect as the reference category. We first ran unadjusted logistic regression analyses for each of the predictors. Next, each predictor was included in an adjusted model to control for the 4 other predictors. The results are presented as un-adjusted and adjusted odds ratios (ORs) with 95% CIs. If a predictor only had a significant contribution in the adjusted model and not in the unadjusted model, a suppressor effect was suspected. Here, a Wald backward stepwise regression procedure (exit criterion *p* = 0.05), starting with all of the predictors in the model, was used to identify the suppressor variables.

## Results

### Study sample

DAWBA interviews were completed for 279 of the 396 eligible children (70.5%), and 175 of these 279 children (62.7%) had information from both a foster parent and a teacher. The DAWBA sections most frequently completed were ADHD (91.0%), ODD/CD (89.6%), and Depression (87.1%), a completion rate in line with previous studies using DAWBA [46].

The child welfare questionnaire was completed for 283 of the 396 eligible children (71.5%). Of the 279 children with DAWBA information, 219 (78.5%) also had information from caseworker questionnaires (See Figure 1).

The calculation of the prevalence of disorders and comorbidity included all of the children with completed DAWBAs (N = 279). Demographic characteristics and associations between possible risk factors and mental disorders were analysed for the subsample with information from both DAWBA and the child welfare questionnaire (n = 219). This subsample and the subsample with only child welfare information (n = 64) showed no significant differences regarding child sex, age, age at first placement, number of former placements, or time in current foster home. No differences between children with DAWBA completed by both carer and teacher (n = 141) and children with DAWBA completed by only one informant were found regarding prevalence of Any disorders, Emotional disorders, ADHD, Behavioural disorders or RAD.

Table 1 shows the demographic characteristics, placement history, and care experiences, as reported by municipal child welfare, of the children with DAWBA and child welfare information (n = 219). According to the information from child welfare, the mean number of aversive childhood experiences before first placement was 3.0 (SD 1.6). Among the children, 42.9% had at least one biological parent born in a non-Western country. Seven children (2.5%) were born outside Norway.

### Prevalence of disorders

Among the 279 children with DAWBA information, a total of 142 children (50.9% CI 45.2–57.0%) met the criteria for at least one DSM-IV disorder (Table 1). Among these, 115 (41.2%) had a disorder in one of the main diagnostic groups: Emotional disorders (24.0%), ADHD disorders (19.0%), or Behavioural disorders (21.5%). The criteria for RAD were met by 19.4%. Of the children aged 6-10 years old (n = 198), where the RAD interview section were included in the DAWBA, 23.2% (n = 46) met criteria for RAD. Additional 4.3% had Pervasive developmental disorders and 2.1% had Tic disorders. No children met the criteria for Panic disorder, Agoraphobia, Selective mutism or Eating disorders (Table 2).

**Table 2:** Point prevalence of DSM-IV disorders in foster children (N = 279)

**DSM-IV Disorder**^**a**^	**n**	**%**	**95% CI**
Any disorder	142	50.9	[45.2, 57.0]
Emotional disorders	67	24.0	[19.0, 29.4]
Separation anxiety disorder	21	7.5	[4.7, 10.8]
Specific phobia	19	6.8	[3.9, 9.7]
Social phobia	3	1.1	[0.0, 2.5]
Posttraumatic stress disorder	14	5.0	[2.5, 7.9]
Obsessive compulsive disorder	1	0.4	[0.0, 1.1]
Generalized anxiety	7	2.5	[1.1, 4.7]
Other anxiety	17	6.1	[3.2, 8.6]
Major depression	3	1.1	[0.0, 2.5]
Other depression	8	2.9	[1.1, 5.0]
Undifferentiated Anxiety/Depression	6	2.2	[0.7, 3.9]
ADHD disorders	53	19.0	[14.7, 24.0]
ADHD Combined	38	13.6	[10.0, 17.9]
ADHD Inattentive	8	2.9	[1.1, 5.0]
ADHD Hyperactive-impulsive	5	1.8	[0.4, 3.6]
Other Hyperactivity NOS	2	0.7	[0.0, 1.8]
Behavioral disorders	60	21.5	[16.8, 26.2]
Oppositional defiant disorder	39	14.0	[10.3, 18.3]
Conduct disorder	18	6.5	[3.6, 9.3]
Other disruptive disorder NOS	3	1.1	[0.0, 2.2]
Reactive attachment disorder	54	19.4	[15.1, 24.0]
Pervasive developmental disorder	12	4.3	[2.2, 6.8]
Tic disorder	6	2.1	[0.7, 3.9]
“Not otherwise specified” disorders	24	8.6	[5.7, 12.2]

^a^No children met criteria for Panic disorder, Agoraphobia, Selective mutism, or Eating disorders.

### Comorbidity

Among the 142 children with mental disorders, 63.4% (90/142) had more than 1 disorder, with a mean of 2.36 disorders (SD 1.52, range 1–7). The rate of comorbidity was 64.2% (43/67) for Emotional disorders, 69.8% (37/53) for ADHD disorders and 81% (49/60) for Behavioural disorders.

Regarding comorbidity between the 3 main diagnostic groups, a total of 30.4% (35/115) had disorders in 2 of the groups, and 13.0% (15/115) had disorders in all 3 groups. The comorbidity between Emotional disorders and either of the 2 other diagnostic groups was 53.7% (n = 36/67). For ADHD disorders, the comorbidity with the 2 other diagnostic groups was 67.9% (n =36/53), whereas the comorbidity rate between Behavioural disorders and the 2 other groups was 71.7% (n = 43/60).

Of the 54 children with RAD diagnoses, a total of 70% (38/54) had at least one comorbid disorder. The comorbidity rate between RAD and the 3 main diagnostic groups was 68.5% (37/54). Thus, only 1 of the children with RAD had a comorbid disorder outside of the 3 main diagnostic groups.

The logistic regression analyses showed that all of the associations between the 3 main diagnostic groups and RAD were significant, except between RAD and ADHD disorders (OR 1.89, CI 0.95-3.77; *p* = 0.070). See Table 3 for details.

**Table 3:** **Odds ratio (and 95**% **Confidence Interval) for comorbid DSM-IV disorders (N = 279)**

	**Emotional**			**ADHD**			**Behavioral**		
	***OR***	**95% CI**	***p***	***OR***	**95% CI**	***p***	***OR***	**95% CI**	***p***
ADHD	2.85	[1.51, 5.39]	.001						
Behavioral	4.46	[2.41, 8.24]	.000	7.60	[3.93, 14.71]	.000			
RAD	3.05	[1.62, 5.74]	.001	1.89	[0.95, 3.77]	.070	6.50	[3.39, 12.67]	.000

Logistic regression analyses run separately for boys and girls, showed that the association between ADHD disorders and Emotional disorders was significant in boys (OR 3.83, CI 1.66–8.87; *p* = 0.002) but not in girls (OR 1.85, CI 0.67–5.08; *p* = 0.235). Furthermore, the association between ADHD disorders and Behavioural disorders was almost twice as high for boys (OR 10.18, CI 4.12–25.20, *p* < 0.001) than for girls (OR 5.41, CI 2.03–14.46; *p* = 0.001), while the association between Behavioural disorders and RAD showed the opposite tendency, with girls having triple the risk of boys (OR 12.40, CI 4.60–33.46; *p* < 0.001) for comorbidity between the 2 disorders (OR 4.23, CI 1.79–10.01; *p* < 0.001).

### Risk factors

The unadjusted and adjusted associations between risk factors and disorders are presented in Table 4. In the unadjusted model, older child age decreased the risk of RAD. After controlling for the other risk factors in the adjusted model, however, this association disappeared. Logistic regression analysis, entering 1 of the other 4 predictors at the same time as age, showed that when controlling for Number of placements, the association between age and RAD became significant. Thus, the effect of age on RAD seemed to be mediated by the number of placements, and age in itself was not a risk factor for RAD.

**Table 4:** Unadjusted and adjusted binary logistic regression analyses of associations between risk factors and disorders (n = 219)

	**Any disorder**		**Emotional disorders**		**ADHD disorders**		**Behavioral disorders**		**RAD**	
**Risk factor**	***OR***	**95% CI**	***OR***	**95% CI**	***OR***	**95% CI**	***OR***	**95% CI**	***OR***	**95% CI**
Age										
Unadjusted	0.99	[0.88, 1.11]	1.06	[0.93, 1.22]	1.07	[0.93, 1.25]	1.01	[0.88, 1.16]	**0.81**	[**0.70, 0.95**]**
Adjusted	1.01	[0.88, 1.16]	1.08	[0.92, 1.27]	1.18	[0.98, 1.42]	1.03	[0.86, 1.23]	0.84	[0.70, 1.02]
Age at first placement										
Unadjusted	0.97	[0.89, 1.07]	1.06	[0.96, 1.17]	**0.84**	[**0.73, 0.96**]**	0.97	[0.87, 1.08]	0.99	[0.89, 1.11]
Adjusted	0.97	[0.87, 1.07]	1.07	[0.96, 1.20]	**0.76**	[**0.64, 0.89**]***	0.88	[0.77, 1.02]	1.00	[0.87, 1.15]
Number of placements										
Unadjusted	0.92	[0.67, 1.25]	1.05	[0.74, 1.50]	**0.38**	[**0.21, 0.67**]***	1.26	[0.88, 1.81]	**1.55**	[**1.08, 2.22**]*
Adjusted	0.91	[0.66-1.26]	1.13	[0.78, 1.63]	**0.30**	[**0.16, 0.58**]***	1.27	[0.86, 1.87]	**1.56**	[**1.06, 2.29**]*
Serious neglect										
Unadjusted	0.82	[0.38, 1.79]	0.75	[0.32, 1.75]	0.48	[0.20, 1.13]	4.10	[0.94, 18.00]	1.30	[0.47, 3.62]
Adjusted	0.84	[0.38, 1.84]	0.70	[0.29, 1.66]	0.45	[0.17, 1.20]	**5.33**	[**1.18, 24.20**]*	1.53	[0.52, 4.48]
Violence exposure										
Unadjusted	1.05	[0.86, 1.30]	0.95	[0.74, 1.21]	1.06	[0.88, 1.37]	**1.35**	[**1.08, 1.70**]**	**1.34**	[**1.06, 1.70**]**
Adjusted	1.05	[0.83, 1.32]	0.86	[0.65, 1.13]	**1.48**	[**1.08, 2.05**]*	**1.64**	[**1.23, 2.18**]***	**1.40**	[**1.06, 1.83**]*

*OR* = odds ratio; CI = Confidence interval; *p < 0.05; **p < 0.01; ***p < 0.001; Significant results are highlighted in bold.

Younger age at first placement increased the risk of ADHD disorders, both in the unadjusted and adjusted analyses.

The number of placements was associated with both RAD and ADHD in unadjusted and adjusted analyses but with opposite effects: A higher number of placements were associated with RAD, whereas lower number of placements was associated with ADHD.

Serious neglect was associated with Behavioural disorders, but only in the adjusted analysis. A backward stepwise (Wald) logistic regression analysis indicated that Violence exposure had a suppressor effect on the association between Serious neglect and Behavioural disorders. After controlling for Violence exposure, Serious neglect increased the risk for Behavioural disorders.

Violence exposure increased the risk for Behavioural disorders and RAD, both in unadjusted and adjusted analyses. Furthermore, Violence exposure also increased the risk of ADHD, but only after controlling for all of the other risk factors in the adjusted model. A backward stepwise (Wald) logistic regression analysis indicated that Age at first placement and Number of placements acted as suppressors on the relationship between Violence exposure and ADHD. After controlling for these 2 risk factors, Violence exposure increased the risk for ADHD.

None of the included predictors was related to the Emotional disorders or Any disorders groups.

## Discussion

### Prevalence of mental disorders

Our findings clearly indicate that foster children constitute a high-risk group for a variety of mental disorders. Our point-prevalence of 50.9% was high, compared to the recent 33.0–38.6% range reported by US and British studies [14,16], and it was closer to the 66.0% rate recently reported for children referred to by specialist mental health services in Norway [47].

Although different diagnostic measurements were used in this study (DAWBA) and in the previous study from the US (DISC) [14], this difference probably does not explain the high prevalence reported here, because DAWBA actually generated lower rates in a direct comparison study that included these 2 measurements [48]. The high prevalence can also not fully be explained by the inclusion of RAD among the diagnoses assessed, because RAD contributed to only 6.1% of the total prevalence in the study sample.

Regarding the main diagnostic groups, the prevalence of ADHD disorders, Behavioural disorders, and Emotional disorders was nearly 10 times greater than what has been reported in epidemiological studies of Norwegian children [32,49]. These 3 main diagnostic groups had fairly equal rates of prevalence in our sample, contrasting the findings from the study of Ford et al. [16], in which Behavioural disorders (32.3%) were more than 3 times more frequent than Emotional disorders (9.7%) and Hyperkinetic disorders (8.5%) [16]. In community samples of children, Behavioural disorders have been found to be more prevalent in the UK than in Norway [50]. One might speculate whether Norwegian children react with more emotional symptoms as a response to neglect and abuse, while British children respond with a stronger tendency to act out. It is also possible that differences in the values, theoretical models and training provided to new foster parents makes Norwegian foster parents more sensitised to emotional symptoms in their foster children than British foster parents.

Our estimated RAD prevalence lies between the prevalence estimate found in a large sample of 6- to 8-year-old, socioeconomically deprived children [51], and the prevalence in severely maltreated toddlers in foster care [52]. Compared to another study on RAD using DAWBA in high-risk foster youths [29], our estimate was quite moderate. Comparisons are difficult, however, due to differences in the criteria used and the sample compositions. Although the overlap between RAD and the other 3 main diagnostic groups was high (68.5%), RAD did not stand out as a disorder with particularly high comorbidity in our study. Thus, our findings contribute to the understanding of RAD among school-aged foster children without institutional backgrounds. However, our present findings should be interpreted with caution and should be validated in other studies including other measurements for assessing RAD.

### Comorbidity among the main diagnostic groups

In the present study, the overall comorbidity among the 3 main diagnostic groups — Emotional disorders, ADHD disorders and Behavioural disorders (43.4%) — was approximately twice as high as that reported for Norwegian children in general [32], and it was even higher than in children referred to specialist mental health services [47]. The high exposure to a broad range of risk factors might, to some extent, explain the differences in comorbidity between foster children and children referred to mental health clinics in general. On average, the children in our study had been exposed to 3 different adverse childhood experiences in their families of origin. Our findings indicate a somewhat different pattern of comorbidity depending on sex, with girls showing a strong overlap between Behavioural disorders and RAD, whereas boys with Behavioural disorders were more likely to have ADHD disorders; however, this difference should be interpreted with caution, due to the small sample size and wide confidence intervals.

Overall, our findings regarding comorbidity highlight the importance of broad assessment approaches covering a wide range of mental health problems to tailor the services addressing the complex symptoms and problem patterns seen among foster children. A recent multilevel meta-analysis on the effects of evidence based treatments, compared to care as usual, indicated that for children and youths with complex, diagnosed disorders, the fixed manual-based treatment had low or non-significant effect sizes [53]. This finding supports the need for treatment planning to be flexible and individually tailored for this high-risk group of children.

### Predictors of mental disorders

It is noteworthy that risk factors only showed associations with externalising and not internalising disorders. Because the Emotional disorders diagnostic group consisted of a total of 10 different single disorders (see Table 2), compared to 3-4 disorders in the two other main diagnostic groups, one could speculate that the former group was too heterogeneous to yield significant results in the analyses of risk factors. However, separate analyses for the two most frequent disorders within the group of Emotional disorders — Separation anxiety and PTSD — yielded the same negative results, indicating that diagnostic heterogeneity cannot explain why Emotional disorders proved to be unrelated to the present risk factors. A recent review of family factors in the development of anxiety disorders concluded that both sexual and physical abuse during childhood appeared to be less strongly linked with anxiety disorders than with other forms of psychopathology, whereas the risk of having an anxiety disorder increased if the parents had anxiety disorders themselves, or the relationship to the child was characterised by overprotection and control [54]. Thus, the content of the child welfare questionnaires might be less relevant for anxiety disorders.

In contrast to previous studies of foster youth [15,16,55] and of Norwegian children in general [32], sex was not related to the prevalence of mental disorders in this study. Our findings are in line with those of McMillen et al.’s study of foster youth [14]. An explanation might be that in samples of children with prolonged exposure to multiple risk factors, the effect of sex will be less pronounced.

Finally, we did not find the increase we expected in mental disorders with increasing age [16]. This finding might be due to the relatively young age and small range of ages in our sample, for which all placements had occurred well before adolescence. Also, additional analysis showed that the association between age and RAD became insignificant when controlling for the number of placements, indicating that it is not age, as such, that is important but the effects of unstable and ruptured attachments.

Somewhat surprisingly, older age at first placement and a higher number of placements decreased the risk for ADHD disorders. This finding might be understood as an ecological correlation, as the temperamental and behavioural problems related to ADHD might increase the probability of parenting problems and thus contribute to early out-of-home placement. It could also be that foster children with ADHD receive more support and have greater access to special education and mental health services, contributing to more stable placements for this group of children. In Norway, it has been documented that children with ADHD disorders have better access to mental health services and special education, compared to children with emotional disorders [32].

Regarding the occurrence of adverse childhood experiences prior to foster placement, Serious neglect was the factor reported most often by the municipalities, with almost 9 out of 10 children having this experience in their family of origin. One might argue that with this very high baseline frequency, this factor lost its predictive power in this sample. Serious neglect was, however, related to an increased prevalence of Behavioural disorders, but only in the adjusted model, in which Violence exposure acted as a suppressor variable.

In our study, Exposure to violence in the family of origin stood out as the most pervasive risk factor for mental disorders, predicting an increased prevalence of 3 of the 4 diagnostic groups: ADHD disorders, Behavioural disorders, and RAD.

It is worth noting that the 4 items of violence exposure included in this factor describe threatening or abusive qualities of the caregiver’s interactions with the child, in which the child’s physical and psychological safety can be seen as endangered by those persons the child depends upon to feel loved and protected. In contrast, parental substance abuse and mental disorders were unrelated to any of diagnostic groups in our sample. Although we were not able to show direct associations between these parental problems and child mental health in this study, parental addiction and mental disorders often co-occur with parental behaviour placing the child`s development in danger, and cannot be ruled out as important risk factors for child development on basis of this study.

### Strengths and limitations

The relatively high overall response rate supports the validity of our findings, although participation bias cannot be ruled out. In other surveys, non-participants have been found to be at higher risk for mental disorders [32], and our estimates might therefore be considered conservative. General strengths of online interviews include ease of participation (not needing to travel or take time off from work for parents), the possibility for obtaining detailed information from multiple informants, and more valid responses to sensitive questions, compared to face-to-face interviews [56]. An obvious strength of the present study was that the information about risk factors and the diagnostic information came from different and independent sources and thus were blinded to each other.

Some of the informants completed only parts of the DAWBA. This limitation might have led to underreporting of disorders. However, according to a recent report form Goodman [57], the completion rate in the present study seemed to be in line with other studies using the DAWBA in epidemiological research. According to Goodman [57], informants primarily completed the sections they identified as relevant to their children and skipped other sections. The high prevalence rate in our study might indicate that foster parents and teachers completed sections that they saw as relevant to the child.

The informants in this study were foster parents and teachers, who are usually aware of the high-risk backgrounds of the children. This fact might have sensitised the informants to look for problems and symptoms in the child, as they know the child has been subjected to neglect and abuse, thus contributing to the high prevalence in this study. However, such an overestimate should then also have been the case for the study of British foster children [16], and it would not explain the discrepancy between the prevalence in these 2 samples.

The present study was based on cross-sectional data, in which placement for at least 5 months was one of the inclusion criteria. The sample might therefore include a disproportionate number of children with long-term placements. In the Norwegian context, this group primarily consists of children with the most severe reasons for placement and thereby represents a high-risk group of foster children. In contrast, national register data indicate that children placed before the age of 13 years old, as in our sample, tend to fare better than those with later out-of-home placements [58]. The young age of the sample might therefore have contributed to a moderate prevalence, which might have been higher had adolescents been included.

Although mean duration of stay in the current foster home was 5.8 years, 23.5% of the children had stayed between five months and two years in their current foster home. In some instances therefore, the foster parents may have limited ability to accurately describe behaviour and emotional development of the child in the DAWBA interview.

Another limitation is the lack of self-report from foster children. This may have led to underreporting of emotional disorders, as they may not be as readily observable by others as behavioural disorders. Further, the study did not include assessment through clinical observation of the children themselves. Still, given the children’s young age and being in a vulnerable position due to problems and conflicts leading to out of home placement, we decided not to interview the children in the study, nor require them to take part in a setting that allowed for direct clinical observation through experts in the area of mental health.

Our study is the first to report on the prevalence of RAD using DAWBA in a sample of school-aged foster children. The fit between the items in the DAWBA RAD section and the DSM-IV criteria has not yet been firmly established. Further studies using this section of the DAWBA are needed to confirm the validity of our findings.

### Clinical implications

Findings have indicated that Norway has a relatively low overall prevalence of child mental disorders [32]. The high prevalence observed in the present study could indicate that the Norwegian welfare-oriented and supportive socioeconomic structure does not offer general buffering effects to this group of children. In contrast, some specific characteristics of the Norwegian child welfare legislation and policies might inadvertently contribute to this high prevalence [3]. Contrary to many other western societies, The Norwegian child welfare-services are not divided into two discrete family-oriented and child-protective services. The child welfare services in Norway, while unifying these two mandates, have traditionally been a distinctly family-oriented service, aiming to support families at risk through preventive and therapeutic programs. Legislation has given priority to interventions within the family before placements out of home are considered. The present study indicates that this family-oriented, partnership approach might need balancing with a stronger child-protection focus, due to the documented detrimental consequences of prolonged exposure to abuse and neglect for the children’s health and development.

Our findings could also shed some light on the reasons for the observed poor effects of traditional mental health services on foster children [59]. High comorbidity and prolonged exposure to a broad range of adverse childhood experiences that are less common among children regularly referred to mental health services might require more specialised mental health services, which are tailored to meet the emotional and practical needs of foster children and their caregivers.

## Conclusions

Our results demonstrated a high prevalence of mental disorders in school-aged foster children, as well as a high rate of comorbidity. The findings also indicated strong associations between indicators of early deficits in care, placement history, and mental disorders. With one in two foster children having a mental disorder; the findings highlight the need for a thorough mental health assessment when a child is placed out of his or her home. To reduce children’s prolonged exposure to adverse childhood experiences, a more child-oriented child welfare policy might need to be considered in Norway.

## Competing interest statement

On behalf of all authors, the corresponding author declares that they have no competing interests.

## Authors’ contributions

SL was responsible for the design of the study, data collection, rating the data, statistical analysis, and manuscript writing. OEH participated in the design of the study, statistical analysis, and commented on the written drafts. TH participated in the design of the study, and commented on the written drafts. ERH participated in the design of the study, rating of data, and commented on the written drafts. All authors read and approved the final manuscript.
